# Illumination of Molecular Pathways in Multiple Sclerosis Lesions and the Immune Mechanism of Matrine Treatment in EAE, a Mouse Model of MS

**DOI:** 10.3389/fimmu.2021.640778

**Published:** 2021-04-12

**Authors:** Mengmeng Dou, Xueliang Zhou, Lifeng Li, Mingliang Zhang, Wenbin Wang, Mengru Wang, Yilei Jing, Rui Ma, Jie Zhao, Lin Zhu

**Affiliations:** ^1^ Department of Pharmacy, the First Affiliated Hospital of Zhengzhou University, Zhengzhou, China; ^2^ Department of Neurology, the Academy of Medical Sciences, Zhengzhou University, Zhengzhou, China; ^3^ Department of Interventional Radiology, the First Affiliated Hospital of Zhengzhou University, Zhengzhou, China; ^4^ Department of Oncology, the First Affiliated Hospital of Zhengzhou University, Zhengzhou, China; ^5^ Internet Medical and System Applications of National Engineering Laboratory, Zhengzhou, China; ^6^ Department of Pharmacy, the first Affiliated Hospital of Henan University of Traditional Chinese Medicine, Zhengzhou, China; ^7^ Department of Pharmacology, School of Basic Medical Sciences, Zhengzhou University, Zhengzhou, China

**Keywords:** matrine, multiple sclerosis, bioinformatics, network pharmacology, IL10RA

## Abstract

The etiology of multiple sclerosis (MS) is not clear, and the treatment of MS presents a great challenge. This study aimed to investigate the pathogenesis and potential therapeutic targets of MS and to define target genes of matrine, a quinolizidine alkaloid component derived from the root of *Sophorae flavescens* that effectively suppressed experimental autoimmune encephalomyelitis (EAE), an animal model of MS. To this end, the GSE108000 gene data set in the Gene Expression Omnibus Database, which included 7 chronic active MS lesions and 10 control samples of white matter, was analyzed for differentially expressed genes (DEGs). X cell was used to analyze the microenvironmental differences in brain tissue samples of MS patients, including 64 types of immune cells and stromal cells. The biological functions and enriched signaling pathways of DEGs were analyzed by multiple approaches, including GO, KEGG, GSEA, and GSVA. The results by X cell showed significantly increased numbers of immune cell populations in the MS lesions, with decreased erythrocytes, megakaryocytes, adipocytes, keratinocytes, endothelial cells, Th1 cells and Tregs. In GSE108000, there were 637 DEGs, including 428 up-regulated and 209 down-regulated genes. Potential target genes of matrine were then predicted by the network pharmacology method of Traditional Chinese medicine, and 12 key genes were obtained by cross analysis of the target genes of matrine and DEGs in MS lesions. Finally, we confirmed by RT-PCR the predicted expression of these genes in brain tissues of matrine-treated EAE mice. Among these genes, 2 were significantly downregulated and 6 upregulated by matrine treatment, and the significance of this gene regulation was further investigated. In conclusion, our study defined several possible matrine target genes, which can be further elucidated as mechanism(s) of matrine action, and novel targets in the treatment of MS.

## Introduction

Multiple sclerosis (MS) is a chronic inflammatory disease of the central nervous system (CNS), characterized by demyelination accompanied by axonal and neuronal degeneration (1, 2). A common disease among young adults, it has affected 2.3 million people worldwide (3, 4). Depending on whether the brain, brain stem, cerebellum, spinal cord of MS patients are affected simultaneously or successively (5), the clinical features and symptoms vary, mainly for limb weakness, paresthesia, decreased vision, ataxia, etc. (6).

The etiology and pathogenesis of MS are not clear, but are most likely due to genetic (e.g. family history) and environmental factors (e.g., viral infection) (7–11). Immune cells play a crucial role in the development of MS, while the specific mechanisms involved need to be further explored. An increased number of IFN-γ^+^ or IL17^+^ CD4^+^ T cells were found in brain and spinal cord lesion tissues in patients with MS, and lymphocytes from relapsing MS patients tended to differentiation of IFN-γ^+^ or IL17^+^CD4^+^ T cells (12). In the cerebrospinal fluid and brain specimens of MS patients, there were IFN-γ^+^ or GM-CSF^+^ CD4^+^ T cells and expression of Th1/Th17-related molecules, namely, chemokine receptors *CXCR3* and *CCR6*, *RORγt* and *T-bet* (13). Further, microglial activation could be found before peripheral infiltration or disease onset of experimental autoimmune encephalomyelitis (EAE), an animal model of MS (14). While different immune cells play their unique roles in MS, traditional detection methods are not capable of simultaneously detecting multiple immune cells. It is therefore important to comprehensively evaluate the importance of immune cells during the pathogenesis of MS.

With the rapid development of microarray and high-throughput sequencing technologies, bioinformatics can be used to evaluate and analyze multiple sets of genome data in multiple paraffin tissues. Therefore, in the public database–GEO data set, we selected the data set according to screening conditions, and applied XCell to evaluate the ratio of 64 immune cells and stromal cells in the CNS lesions of MS patients through the specific algorithm inference. The biological functions and enriched signaling pathways of differentially expressed genes (DEGs) were analyzed by GO, KEGG, GSEA, GSVA. Further, possible target genes of a novel treatment could be predicted based on the network pharmacology of Traditional Chinese medicine. Matrine, a quinolizidine alkaloid component derived from the root of *Sophorae flavescens*, has had antibacterial and anti-inflammatory effects in clinical practice, as well as in the treatment of hepatitis B and C (15–18). We have previously reported that MAT decreased clinical EAE severity, inhibited production of inflammatory cytokines/chemokines, induced anti-inflammatory molecules such as IL-4 and IL-10 (19) and promoted regeneration of injured myelin sheaths by inhibiting the pathway of nerve regeneration induced by Nogo-A (20). However, the mechanisms underlying these effects are still unknown. To bridge this gap, we conducted cross analysis between DEGs in MS patients and the predicted target genes of matrine treatment in EAE mice to explore the possible mechanisms of matrine action on EAE, and likely on MS as well.

## Materials and Methods

### Microarrays Datasets Collection and Data Preprocessing

Gene expression data sets were from GEO Data Sets (https://www.ncbi.nlm.nih.gov/). We selected the potential GEO data sets based on the following search terms: “Homo sapiens” (Organism) and “Multiple Sclerosis” (MeSH Terms) and “Expression profiling by array” (Data Set Type). Data sets from other biological samples such as mice were excluded. We then eliminated data sets from blood samples or immune cells and got the GSE108000 data set, which included 7 chronic active MS lesions and 10 control white matters. The corresponding platform annotation file (GPL13497) was used to convert the probe number in the data set into the gene name. If a probe corresponded to multiple genes or there was no corresponding gene to the probe, these probes were removed. If multiple probes corresponded to the same gene, the average expression value of multiple probes was taken as the expression value of the gene. We used the “Normalize Between Arrays” in the R “LIMMA” package (http://www.bioconductor.org/) to standardize the raw data of quantile.

### DEGs Analysis and Immune and Stromal Cells Analysis

DEGs analysis was performed by the R package “LIMMA.” The Benjamini & Hochberg error discovery rate was used to perform multiple corrections on the original P value, and the adjusted P value was obtained. The Fold Change (FC) of gene expression in white matter and chronic active MS lesions was calculated. For the identification of DEGs, the cut-off criteria of |log_2_ Fc| value was more than 1 and the adjusted P value was less than 0.05. By applying Xcell (http://xCell.ucsf.edu/) to the microarray data and the Wilcoxon variance methods, we were able to obtain an estimated proportion of the immune cell and stromal cell types in each brain tissue sample (p<0.05). Sixty-four cell types are categorized into immune cells, hematopoietic stem cells (HSCs), stroma and epithelial cells, etc. (21).

### Functional Enrichment Analysis of DEGs

We used the Gene Ontology (GO) database (http://www.geneontology.org), which includes biological process (BP), cell component (CC) and molecular function (MF), to analyze possible protein functions of DEGs. The Kyoto Encyclopedia of Genes and Genomes (KEGG) database (http://www.kegg.jp/) was used to predict the signaling pathways involved in the DEGs. Fisher’s exact test and the multi-comparison test were used to calculate the significance level of each function or signaling pathway (P < 0.05).

### Protein-Protein Interaction Network Analysis

We used the online database STRING (http://string-db.org/) for protein-protein interaction (PPI) network analysis. We mapped DEGs onto the PPI network, and the interaction score of more than 0.9 was set as the threshold. The data obtained from the STRING database were imported into Cytoscape (V3.7.2) software. Degree algorithm of CytoHubba was then used to screen the key genes from the network. The top 20 genes rated by the algorithm were the key genes.

### Gene Set Enrichment Analysis and Gene Set Variation Analysis

We used Gene Set Enrichment Analysis (GSEA) software (22) to assess differentially enriched pathways between MS patients and control groups and to identify the rich immune-related signaling pathways. We then used Gene Set Variation Analysis (GSVA), which is a GSE method for analyzing data sets of KEGG enriched pathways (23). In this study, we used the R package “GSVA” to calculate the scores of each patient based on a predefined set of KEGG path genes. A linear model was then modeled by using R-pack LIMMA to compare GSVA scores between MS patients and control groups, and the significantly changed paths were defined with P <0.05 and |log_2_ FC|≥0.2. Speedman-related analysis and linear regression analysis of the correlation between immune cells and KEGG key paths were carried out.

### The Drug-Target Prediction of Oxymatrine by Chinese Medicine Network Pharmacology

Based on the theory of system biology, network pharmacology analyzes the network of biological systems and selects specific signal nodes for multi-target drug molecular design. Network pharmacological analysis can improve the therapeutic effect of drugs and reduce toxic side effects, thus improving the success rates of clinical trials of new drugs and saving the cost of drug research and development. The 2D and 3D structure of Oxymatrine and Oxysophocarpine was searched on the website https://pubchem.ncbi.nlm.nih.gov/. We then uploaded the 3D structure of Oxymatrine to the Pharmmapper database (http://www.lilab-ecust.cn/pharmmapper/) to predict possible drug targets. Based on the predicted results, we performed further analyses, namely PPI analysis, GO function analysis and KEGG signaling pathway analysis (as previously described).

### Induction and Matrine Treatment of EAE

C57BL/6 female mice, 8-10 weeks old and weighing 20 ± 2g, were purchased from Beijing Weitonglihua Experimental Animal Technology Company, and raised in an SPF-class animal room at the Experimental Animal Center of Zhengzhou University. Heat-killed mycobacterium tuberculosis (100 mg) was added into 10 ml Freund’s adjuvant, fully dissolved, and mixed thoroughly with an equal volume of MOG35-55 (2 mg/ml) to prepare a water-in-oil antigen emulsion. Mice anesthetized with isoflurane were injected with the emulsion at three points along the upper and lower sides of the midline, a total of 200 μl/mouse. On days 0 and 2 post immunization (p.i.), 200 ng pertussis toxin was injected intraperitoneally. Mice were weighed daily and, at the onset of disease, clinical scores were recorded following a 0-5 scale (24): 0 – normal; 1 - weakness of tail; 2 - moderate weakness of forelimbs or hindlimbs; 3 - severe weakness of forelimbs or hindlimbs; 4 - limb paralysis, the mice could not turn back over after artificial overturning; 5 -death. The above trials were repeated twice to ensure the reproducibility of clinical efficacy. Mice were randomly divided into three groups (n = 10 each group), and treatments included: 1) Saline: mice received 20 ml/kg per day normal saline i.p. from day 7 p.i.; 2) MAT-L: mice were injected i.p. with 100 mg/kg (20 ml/kg) per day of MAT (Jiangsu Chia Tai Tianqing Pharmaceutical Co., Jiangsu, China); and 3) MAT-H: mice were injected i.p. with 200 mg/kg (20 ml/kg) per day of MAT. The drug doses were referenced as previously described (24), and treatments were started from day 7 p.i. daily up to day 23 p.i. In addition, 6 normal mice were used as naïve control. All experimental procedures were approved by the Ethics Committee of Zhengzhou University.

### H&E and Luxol Fast Blue (LFB) Stainings

H&E staining was used to evaluate the levels of inflammatory cell infiltration. Under the optical microscope, 5 to 10 fields (×200) were randomly selected from each section to observe the infiltration of inflammatory cells. The following criteria were used for scoring: 0 point, no inflammatory cells were found; 1 point, a small number of scattered inflammatory cells were found; 2 points, inflammatory cells around the blood vessels were found to infiltrate into the cuff; 3 points, a large number of vascular cuff lesions were found. LFB staining was used to evaluate the levels of demyelination. Under the optical microscope, 5 to 10 fields (×200) were randomly selected from each section to observe and analyze the situation of demyelination. The score was recorded according to the following criteria: 0, no demyelination; 1, rare demyelination; 2, a few demyelinated areas; 3, large area of demyelination.

### RNA Extraction and qRT -PCR

Total RNA was extracted from tissues using TRIzol reagent (Invitrogen, USA), and RNA concentration and purity were detected by a UV spectrophotometer. A cDNA kit (Thermo, USA) was used for reverse transcription. Specifically, genomic DNA was removed at 42°C for 2 minutes, and the reverse transcription reaction was performed at 37°C for 15 minutes and 85°C for 5 seconds. Using cDNA as the template, PCR was performed with FastStart Universal SYBR Green Master Mix (Roche, USA). GAPDH was used as an internal control. The primer sequences of target genes are shown in **Table 1**.

**Table 1:** Primers and expected sizes of PCR products of target genes.

Gene	Sense Primers (5’-3’)	Anti-sense Primers (5’-3’)	Product size (bp)
ANG	TTCGTGCTGGGTCTGGTTGT	CAGGGTGAGGTTAGGCTTCTTCT	155
SFN	GGGAGAAGGTAGAGACCGAGCT	GCTATCTCGTAGTGGAAGACTGAAA	298
DAAM1	CGGCTTAGGAATGATAGCAACTT	ACTGGTTGCTCCCTTGTTTTCT	219
DPP4	TCATCACCGTGCCAATAGTTCT	TCTGAAACCCACCACAAAGAGTAG	130
FABP7	GGGAAACGTGACCAAACCAA	CCAACCGAACCACAGACTTACA	164
IL10RA	CACCCTGGATCTGTATCACCG	TAGATGATGCCGTCCATTGCT	165
MERTK	CCCGCCCACTGAAGTCCATA	GAGCCATTACTCAGCCGGTCA	138
MSN	GACTTGGAGAAGACTCGTGCTG	TCTTGGACTCATCTCGGGCAT	250
C3	AAACTTCGGGGAAACGGTG	CCCACGGGCAGTAGGTTGTT	150
GAPDH	CCTCGTCCCGTAGACAAAATG	TGAGGTCAATGAAGGGGTCGT	133

### Statistical Analyses

The statistical analysis was carried out by R software and GraphPad Prism7.0 (GraphPad Software, Inc., La Jolla, CA, United States), and the grade correlation between different variables was evaluated by Pearson correlation coefficient. Multiple comparisons performed by ANOVA were used to analyze the weight changes and the difference in mean clinical severity of mice. The Student’s *t* test was used to analyze H&E staining and LFB staining scores. All values are presented as mean ± SD. P<0.05 was considered statistically significant.

## Results

### Analysis of Immune Cells and Stromal Cells

**Figure 1:**
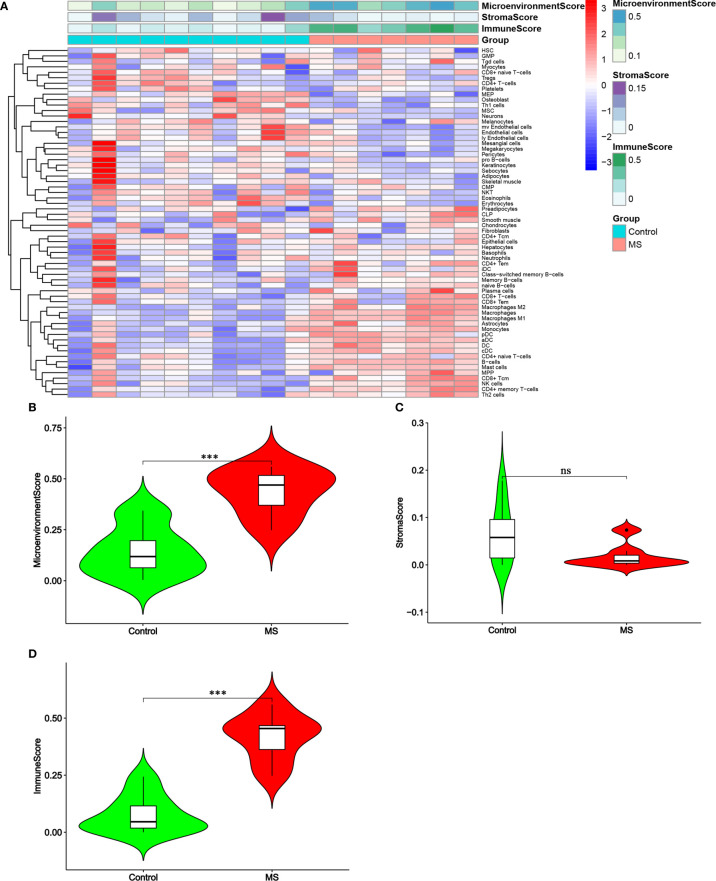
Distribution of immune cells and stromal cells in MS and control groups. **(A)** Heatmap of normalized absolute abundance for each cell type in every single sample. **(B–D)** Micro-environment Score, Stroma Score and Immune Score of the samples. Green represents the control group and red represents the MS group. ***P < 0.001. ns, not significant.

The raw data included 7 chronic active MS lesions and 10 control white matters. Following the algorithm described previously (21), the heat map showed the distribution of hematopoietic stem cells (HSC), stroma and epithelial cells, and immune cells in the brain tissues of two groups (**Figure 1A**). Significantly higher Microenvironment Score and Immune Score, but not Stroma Score, were shown in MS lesions than in control (P<0.01; **Figures 1B–D**).

**Figure 2:**
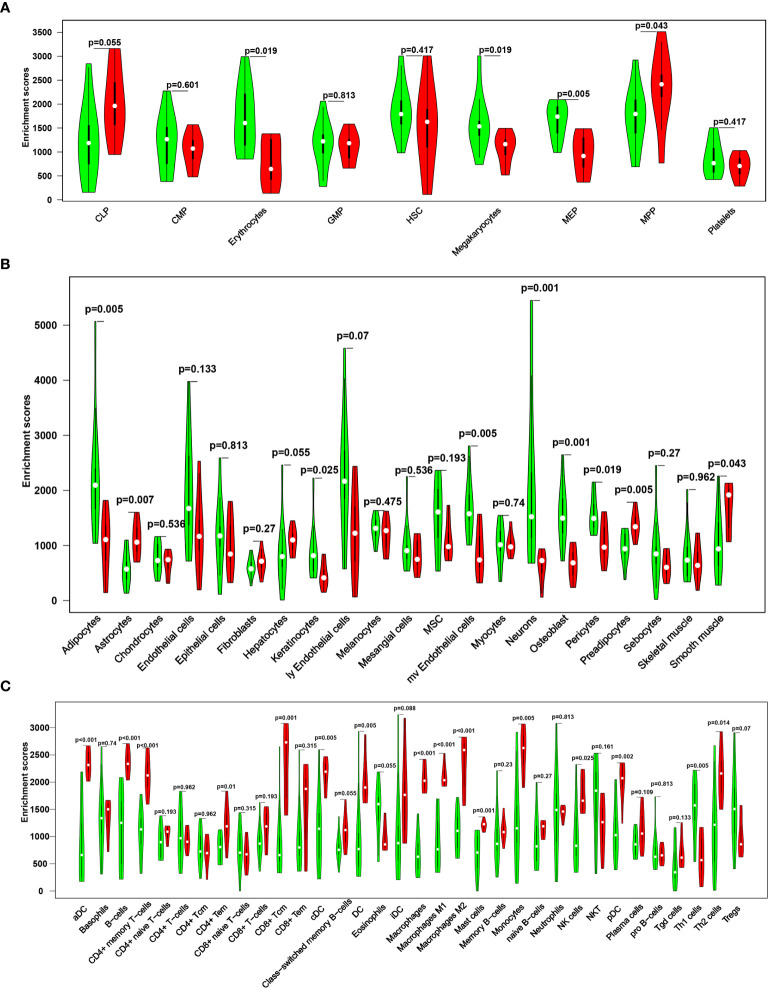
A violin diagram comparing the proportion of hematopoietic stem cells **(A)**, stroma and epithelial cells **(B)**, and immune cells **(C)** in the brain tissues of two groups. Green represents the control group, and red represents the MS group. P < 0.05 was considered statistically significant.

By comparing the scores of multiple cell types (**Figures 2A–C**), the results showed that, compared with the control group, MS group showed significantly decreased scores for erythrocytes, megakaryocytes (HSC), Adipocytes, keratinocytes, mv endothelial cells, neurons, osteoblasts, pericytes (stroma and epithelial) and Th1 cells (immune cells) (P<0.05). In contrast, the MS group showed significantly increased scores for MPP (HSC), astrocytes, preadipocytes (stroma and epithelial), aDC, B cells, CD4+ memory T cells, CD4+ Tem, CD8+ Tcm, cDC, DC, M1/M2 macrophages, mast cells, monocytes, NK cells, pDC, and Th2 cells (immune cells) (all P<0.05).

### Screening and Biological Function Analysis of DEGs

**Figure 3:**
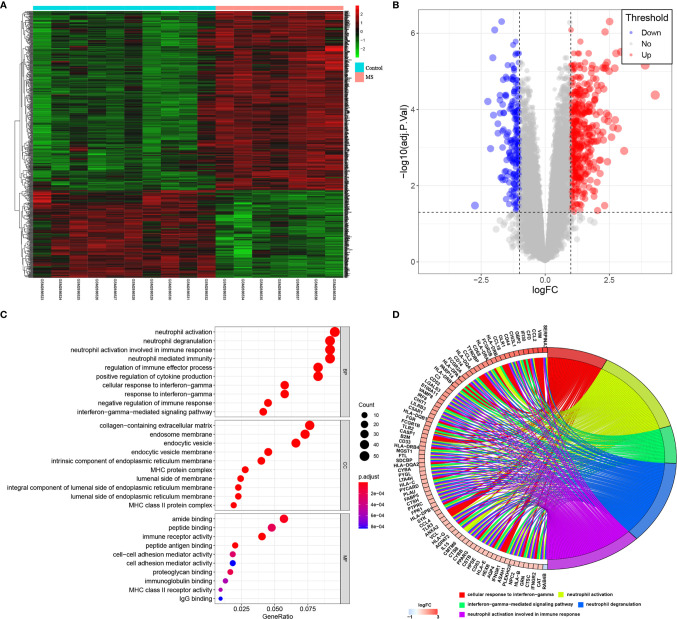
GSE108000 screening of DEGs and GO analysis. **(A, B)** Heatmap and Volcano Plot of DEGs in MS and control groups. **(C, D)** The distribution of DEGs in different GO functions, namely, the biological process (BP), cell component (CC) and molecular function (MF).

By GSE108000 screening, 637 DEGs, including 428 up-regulated genes and 209 down-regulated genes were identified, as shown in **Figures 3A, B**. We then analyzed these genes based on their biological functions. **Figures 3C, D** shows the distribution in different GO functions, namely biological process, cell component and molecular function. Top 10 biological functions were selected in each section. For biological process, the DEGs were mainly related to neutrophil activation, neutrophil degranulation, neutrophil activation involved in immune response, neutrophil-mediated immunity, regulation of immune effector process, positive regulation of cytokine production, cellular response to interferon-gamma, negative regulation of immune response, and interferon gamma-mediated signaling pathway. For cell component, DEGs were associated with the processes of collagen-containing extracellular matrix, endosome membrane, endocytic vesicle, endocytic vesicle membrane, intrinsic component of endoplasmic reticulum membrane, MHC protein complex, lumenal side of membrane, integral component of lumenal side of endoplasmic reticulum membrane, lumenal side of endoplasmic reticulum membrane, and MHC class II protein complex. For molecular function, this involved tamide binding, peptide binding, immune receptor activity, peptide antigen binding, cell-cell adhesion mediator activity, cell adhesion mediator activity, proteoglycan binding, immunoglobulin binding, MHC class II receptor activity, and IgG binding. Together, the analysis showed that these important differentially expressed genes were involved in the innate immune response.

### Pathway Enrichment Analysis by KEGG

**Figure 4:**
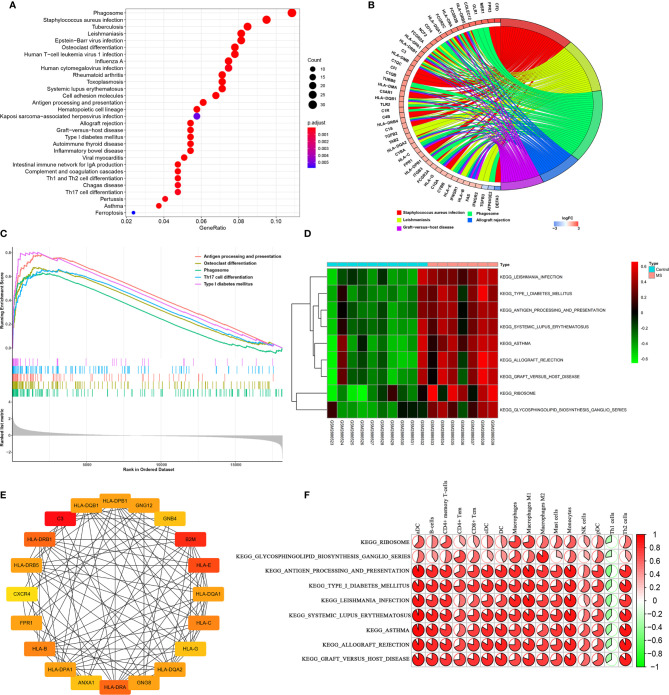
Enrichment analysis and correlation analysis of DEGs in the KEGG pathway. **(A, B)** The distribution of DEGs in the KEGG pathway. **(C, D)** GSEA and GSVA for significantly enriched pathways in MS and control groups. **(E)** Hub genes with the top 20 degrees of interaction were identified using Cytohubba. **(F)** Correlation analysis based on Pearson r value between immune cells and KEGG key pathways. P<0.05 and |log2FC|≥0.2 were defined as significant changes.

We then analyzed pathway enrichment by KEGG, and the DEGs results are shown in **Figures 4A, B**. The pathways include Staphylococcus aureus infection, Leishmaniasis, Phagosome, Allograft rejection, Graft-versus-host disease, Type I diabetes mellitus, Rheumatoid arthritis, Autoimmune thyroid disease, Osteoclast differentiation, Antigen processing and presentation, Toxoplasmosis, Inflammatory bowel disease, Intestinal immune network for IgA production, Viral myocarditis, Asthma, Tuberculosis, Systemic lupus erythematosus, Hematopoietic cell lineage, Influenza A, Epstein-Barr virus infection, Cell adhesion molecules, Complement and coagulation cascades, Human T-cell leukemia virus 1 infection, Th1 and Th2 cell differentiation, Pertussis, Human cytomegalovirus infection, Kaposi sarcoma-associated herpes virus infection, and Ferroptosis. GSEA software was then used to assess differences between MS and control groups in enrichment pathways, and the gene sets c2.cp.kegg.v7.1.symbols.gmt previously annotated was selected as the internal gene list (**Figure 4C**). In addition, we conducted GSVA to assess potential changes in pathway activity, and the R-package “GSVA” was used to calculate the scores of each sample based on the gene set of the previously defined KEGG pathway. Subsequently, we used R package limma to establish a linear model to compare GSVA scores between MS and control groups. P<0.05 and |log_2_FC|≥0.2 were defined as significant changes in the pathway (**Figure 4D**). The results of PPI network construction for DEGs are shown in **Figure 4E**. The top 20 hub genes for Degree Algorithmic Scoring were: HLA-DQB1, HLA-DPB1, GNG12, GNB4, B2M, HLA-E, HLA-DQA1, HLA-C, HLA-G, HLA-DQA2, GNG8, HLA-DRA, ANXA1, HLA-DPA1, HLA-B, FPR1, CXCR4, HLA-DRB5, HLA-DRB1, and C3. We then performed spearman correlation analysis and linear regression analysis on the correlation between immune cells and KEGG key pathways, as shown in **Figure 4F**. These key DEGs were positively correlated with the antigen processing and presentation, systemic lupus erythematosus, graft vs. host disease pathway of B cells, DC, macrophages and Th2 cells, but negatively correlated with Th1 cells. Together, the analysis showed that these differentially expressed genes might lead to the progression of MS by influencing immune cells and mediating several key pathways of these immune cells.

### Target Genes Prediction of Matrine Injection

**Figure 5:**
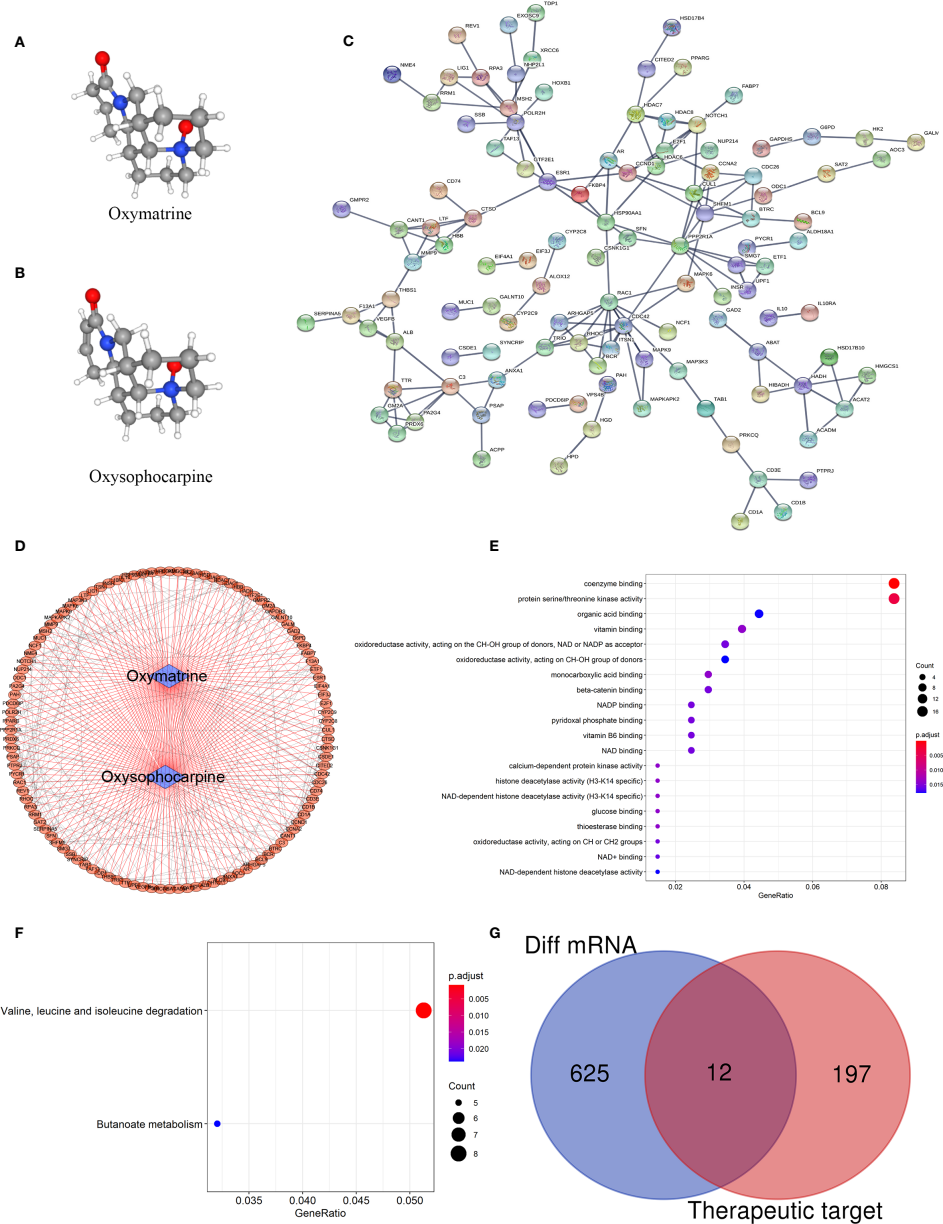
Target gene prediction of matrine and mechanism of matrine to MS. **(A, B)** 3D structure of oxymatrine and oxysophocarpine. **(C, D)** PPI network of target genes. **(E)** Top 20 GO function analysis of target genes. **(F)** KEGG signaling pathways of target genes. **(G)** Venn diagram of DEGs among MS and control brain tissues (the blue part) and target genes of matrine (the red part).

The main components of the matrine injection are oxymatrine and oxysophocarpine. Matrine has antibacterial and anti-inflammatory effects, and is used to treat hepatitis B and C. Recent studies (20, 25) have found that matrine has immunomodulatory effects and has benefits for autoimmune diseases. Based on the network pharmacology of Traditional Chinese Medicine, we predicted the possible targets of matrine. The 3D structure of oxymatrine and oxysophocarpine were as shown in **Figures 5A, B**. By matching the 3D structures of oxymatrine and oxysophocarpine with the Pharmapper database, we found that there were 205 targets for oxymatrine and 203 for oxysophocarpine. More interestingly, they have 199 overlapping target genes. The PPI network and biological functions of these target genes were further analyzed (**Figures 5C–F**) and were found to be mainly concentrated in the following functions: coenzyme binding, protein serine/threonine kinase activity, organic acid binding, vitamin binding, oxidoreductase activity, acting on the CH-OH group of donors, NAD or NADP as acceptor, oxidoreductase activity, acting on the CH-OH group of donors, monocarboxylic acid binding, beta-catenin binding, NADP binding, pyridoxal phosphate binding, vitamin B6 binding, NAD binding, histone deacetylase activity (H3-K14 specific), NAD-dependent histone deacetylase activity, glucose binding, thioesterase binding, oxidoreductase activity, acting on the CH-OH group of donors, NAD or NADP as acceptor, NAD+ binding, and NAD-dependent histone deacetylase activity. The KEGG signaling pathways were mainly Valine, leucine and isoleucine degradation and Butanoate metabolism. Finally, we analyzed the crossover between DEGs analyzed previously and the target genes of oxymatrine and oxysophocarpine to predict the possible mechanism of matrine injection to MS, and twelve genes were found to overlap between MS tissues and matrine (**Figure 5G**). These genes could be potential target genes for matrine treatment in EAE subject to confirmation of efficacy and further study.

### The Therapeutic Effects of Matrine Injection on EAE Mice

**Figure 6:**
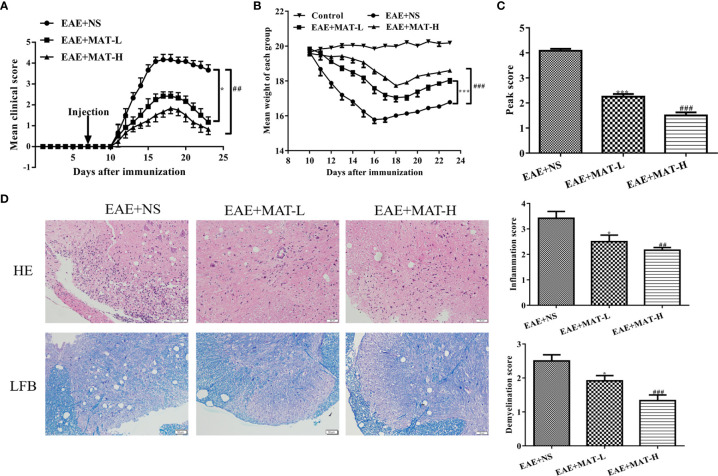
Matrine treatment reduced EAE severity, CNS inflammatory infiltration and demyelination. EAE was induced in C57BL/6 mice by MOG35-55/CFA/PTX as described in Materials and Methods. Starting from day 7 p.i., 0.1 ml MAT at 100 mg/kg (MAT-L) or 200 mg/kg (MAT-H) was i.p. injected daily, and the control mice were given the same volume of normal saline (NS). Mean clinical score **(A)**, body weight **(B)** and peak score **(C)** of EAE+NS, EAE+MAT-H, EAE+MAT-L and naïve control group. The lumbar enlargement of the spinal cord was harvested on day 23 p.i. after heart perfusion. H&E staining was used to evaluate the levels of inflammatory infiltration, and LFB staining to evaluate the levels of demyelination. **(D)** H&E and LFB staining of the spinal cord in all groups. * or *** represents the comparison between EAE+NS and EAE+MAT-L groups, and ## or ### represents the comparison between EAE+NS and EAE+MAT-H groups. Data are shown as mean ± SD (n=10 each group). * P < 0.05; ## P < 0.01; *** or ### P < 0.001.

In order to analyze whether the defined matrine target genes are indeed involved in its therapeutic effects on EAE, C57BL/6 mice were immunized with MOG35-55/CFA/PTX. Matrine was i.p. injected with low (100 mg/kg) or high (200 mg/kg) doses daily, starting from day 7 p.i. Saline-treated control mice had the first signs of EAE on day 11 p.i., and the disease reached its peak on day 16 p.i., with reduced body weight. Mean clinical scores were significantly reduced and body weight was higher in the MAT-treated group compared to the saline-treated group in a dose-dependent manner (**Figures 6A–C**). Mice were then sacrificed at day 23 p.i., and the brain and spinal cord were harvested after heart perfusion. HE staining and LFB staining of the spinal cord showed an extensive infiltration of inflammatory cells, perivascular cuffing and large areas of demyelination in the saline-treated group, while MAT treatment significantly inhibited the infiltration of inflammatory cells into the CNS of EAE mice, with decreased demyelination. (**Figure 6D**).

### Confirmation of Genes Predicted in Brain Tissues of Matrine-Treated EAE Mice

**Figure 7:**
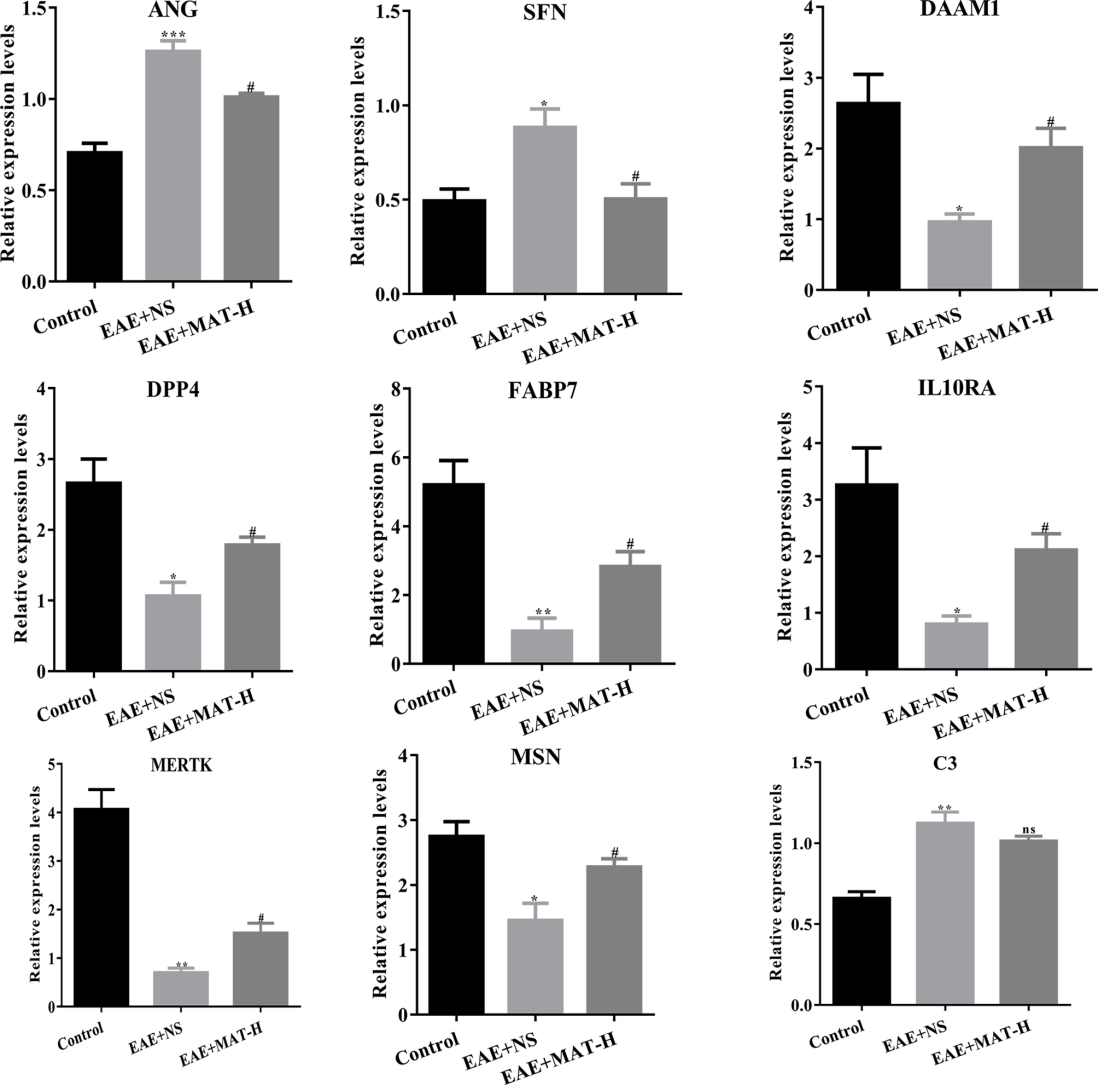
The relative expression of key genes by qPCR. ANG, SFN, DAAM1, DPP4, FABP7, IL10RA, MERTK, MSN and C3 of naïve control, EAE+NS, EAE+MAT-H groups. On day 23 p.i., the brain tissues of mice were harvested after heart perfusion. qPCR was used to evaluate the relative expression levels of key genes in each group. *, ** or *** represents the comparison between naïve control and EAE+NS groups, and # represents the comparison between EAE+NS and EAE+MAT-H groups. Data are shown as mean ± SD (n=6 in Control, n=10 in each EAE group). *or # P < 0.05; ** P < 0.01; *** P < 0.001. ns, not significant.

By cross analysis of the DEGs in MS lesions and target genes of matrine injection, 12 key genes were obtained, namely Annexin A1 *(ANXA1)*, Fatty acid binding protein 7 *(FABP7)*, Moesin *(MSN)*, Glycogen phosphorylase L *(PYGL)*, Dipeptidyl peptidase 4 *(DPP4)*, complement C3 *(C3)*, Peroxisome proliferator activated receptor gamma *(PPARG)*, MER proto-oncogene, tyrosine kinase *(MERTK)*, IL-10 receptor subunit alpha *(IL10RA)*, Stratifin *(SFN)*, Angiogenin *(ANG)*, and dishevelled-associated activator of morphogenesis 1 *(DAAM1)*. To validate these findings and further explore the mechanism of matrine treatment in EAE mice, brain tissues of EAE mice treated with matrine and saline control were harvested, and their mRNA expression of these 12 genes was determined. The expression of *ANG* and *SFN* in the CNS of saline-treated EAE mice was increased compared with that in normal control mice, while their expression was significantly reduced after matrine treatment. In contrast, the CNS of saline-treated EAE mice exhibited decreased expression of *DAAM1, DPP4, FABP7, IL10RA, MERTK* and *MSN* compared with that in normal control mice, and their expression was significantly up-regulated by matrine treatment. While the CNS of saline-treated EAE mice exhibited increased expression of C3, matrine treatment did not significantly change its expression (**Figure 7**). There were no significant differences observed for *ANXA1, PYGL* and *PPARG* among these three groups (data not shown).

## Discussion

MS, an autoimmune demyelinating disease of the CNS, is the main cause of disability except injuries in young adults. Treatment at the acute stage is mainly to relieve symptoms and reduce the degree of disability as much as possible, while treatment at the remission stage is mainly to reduce the recurrence of brain and spinal lesions, to delay the accumulation of disabilities and to improve the quality of life. However, these disease–modifying therapies have a series of side effects. The use of glucocorticoids, for example, has caused muscle atrophy (26), osteoporosis, obesity, cataracts, necrosis of the femoral head and immunosuppression (27), while IFN-β and natalizumab were respectively predicted to have 62% and 80% annual recurrence and 2-year disability deterioration in MS patients receiving their first treatment (28, 29). The development of a new, personalized medicine is therefore urgent.

Matrine injection has a clear therapeutic effect on viral hepatitis (30), and also regulates the number and function of leukocytes (31). Our previous studies have shown that matrine treatment inhibited inflammatory but induced immunomodulatory molecules (32–35), protected the integrity of blood-brain barrier (36), and promoted neural repair or protected neural cells from inflammation-induced cell death (37–39). To further explore the molecular mechanism underlying these effects, we in the present study used TCM network pharmacology analysis to predict the target genes of matrine injection, and bioinformatics to analyze DEGs among MS patients and controls. Indeed, the development of bioinformatics provides new methods to study the specific mechanism of drug therapy for diseases, and these novel technologies have been used for new understanding for the treatment of diseases with Traditional Chinese medicine (40). Our results showed that the main components of matrine, oxymatrine and oxysophorine, had 199 target genes overlapped, which were mainly enriched in 25 functions, such as protein serine/threonine kinase activity and the enrichment pathways were valine, leucine and isoleucine degradation and Butanoate metabolism. In combination with the DEGs from CNS lesions of MS patients analyzed in the GSE108000 data set, a total of 12 key genes were found. These results were further confirmed in the CNS tissues of EAE mice after matrine treatment, which effectively suppressed clinical severity and CNS inflammatory demyelination. The expression of the majority of these genes predicted had been altered in EAE, either up- or down-regulated by matrine treatment. Thus, cross analysis of DEGs in MS lesions with the network pharmacology method of Traditional Chinese medicine could be a powerful approach to predict key target genes and the mechanism of action of a medication of interest.

Among the key genes predicted, two were significantly enhanced in the CNS of saline-treated EAE mice, and were inhibited by matrine treatment, including angiogenin (ANG) and Stratifin (SFN). ANG encoded proteins have been shown to be neurotrophic and neuroprotective, with antimicrobial effects (including bacteria and fungi). ANG I therapy could alleviate the blood-brain barrier leakage caused by inflammation, inhibit the infiltration of inflammatory cells into the brain and spinal cord, and improve EAE-related dysfunction in a dose-dependent manner (41). In contrast, ANG II is a powerful pro-inflammatory mediator and plays an important role in autoimmune diseases including rheumatoid arthritis (RA), systemic lupus erythematosus and MS (42). Oral candesartan, an ANG II receptor antagonist, significantly reduced optic neuritis and spinal cord demyelination in EAE mice at the early stage, and reduced retinal ganglion cell loss and visual impairment (43). SFN, also known as 14-3-3 protein, encodes a cell cycle checkpoint protein, which plays a role in preventing DNA errors during mitosis. SFN is abundant in neurons, and its abnormal expression and impaired function have been associated with the pathogenesis of Alzheimer’s disease, Parkinson’s disease, and MS (44). The regulatory region of SFN gene has two sets of common sequences that bind p53, which is the central regulator of DNA damage response (45). Satoh et al. found that *in vivo*, reactive astrocytes in MS demyelinating lesions expressed SFN, and this was associated with increased oxidative stress and DNA damage response (46). These results, together with our data, indicate a proinflammatory role of ANG and SFN in CNS autoimmunity, and their inhibition could be a mechanism of matrine action in the therapy of EAE.

In contrast, 6 genes among the key genes predicted were significantly reduced in the CNS of saline-treated EAE mice, and were upregulated by matrine treatment, thus implying an immunomodulatory role in EAE. Indeed, among them, IL-10RA is structurally related to interferon receptor, which mediates the immunosuppressive signal of IL-10 and inhibits the synthesis of proinflammatory cytokines (47). It has been found that Tr1 cells isolated from MS patients produced less IL-10 than those obtained from controls, and the activity of IL-10R signaling and IL-10 regulatory function is impaired in patients with MS (48). IL-10 signaling is essential for 1,25-dihydroxyvitamin D3-mediated inhibition of EAE (49). Type I and III interferons enhance IL-10R expression on human monocytes and macrophages, resulting in IL-10-mediated suppression of TLR-induced IL-12 (50). Consistent with these observations, in the present study we showed significantly upregulated IL-10R after matrine treatment, indicating an increased IL-10 responsiveness. This result, together with our previous observation for an increased IL-10 production after matrine treatment (19), provide solid evidence for the important role of the IL-10/IL-10R system in the therapeutic effect of matrine. Similarly, fatty acid binding protein 7 (FABP7) encodes a small but highly conserved cytoplasmic protein, which can bind long-chain fatty acids and play an important role in the establishment of the radial glial fiber in brain. Expression of FABP7 in activated astrocytes is related to early remyelination (51). Upregulation of FABP7 could therefore be beneficial for EAE treatment by matrine. Further, tyrosine kinase (MERTK), an MER proto-oncogene, is a member of the MER/AXL/TYRO3 receptor kinases family that can scavenge apoptotic cells to maintain homeostasis. Defective MERTK expression leads to chronic inflammation and autoimmune diseases such as atherosclerosis, MS, systemic lupus erythematosus, rheumatoid arthritis and Crohn’s disease (52). Single nucleotide polymorphisms (SNPs) in MERTK also leads to a range of chronic inflammatory and autoimmune diseases, most notably, atherosclerosis and MS (53, 54). Moreover, changes in MERTK might be related not only to MS susceptibility, but also to disease progression (53). Dishevelled-associated activator of morphogenesis 1 (DAAM1) encodes a membrane-localized protein that binds phospholipids. Dipeptidyl peptidase 4 (DPP4) encodes dipeptidyl peptidase 4, which is equal to adenosine deaminase complexing protein-2, and to the T cell activation antigen CD26. DDP4 is highly correlated with glucose and insulin metabolism, and also with immune regulation. Moesin (MSN) includes Ezrin and Radixin, which are important for cell recognition, signal transduction, and cell movement, and have also been associated with promoting T cell activation and polarity (55). Thus, our data, together with the literature, indicate that upregulation of IL-10R, FABP7, and MERTK could be a mechanism of matrine treatment in EAE. While there have been no previous reports showing an immunomodulatory role of DAAM1, DPP4 or MSN, our results imply this possibility, which needs further investigation.

Lastly, there were a few genes predicted to be involved in MS pathogenesis and in matrine treatment, but which could not be confirmed. Among them, Complement component C3 is important in the activation of the complement system. C3a peptide can regulate inflammation and has antibacterial activity. It was found that C3-RS2230199, a common encoding variant of the C3 gene, affected white matter and gray matter damage and cognitive impairment in MS patients (56). Annexin A1 (ANXA1) encodes a membrane-localized protein that can bind phospholipids. This encoded protein can inhibit the activity of phospholipase A2 and has an anti-inflammatory effect. ANXA1 was also considered as a primary endogenous mediator of the anti-inflammatory and immunosuppressive actions of gluco-corticoids (57). Peroxisome proliferator activated receptor gamma (PPARG) encoded a protein called PPARγ, which regulates adipocyte differentiation and has been implicated in the pathology of many diseases, including obesity, diabetes, atherosclerosis and cancer (58–60). Ursolic acid has been reported to play an anti-inflammatory and neurorepair role by upregulating myelin-related gene expression through PPARγ activation (61). While these molecules could be of interest in general, they may not be involved in the effect of matrine treatment in EAE in particular.

In summary, by conducting cross analyses between DEGs in MS patients and the predicted target genes of matrine treatment in EAE mice, our present study uncovered interesting candidates, both neuroprotective and immunomodulatory molecules, which can be further elucidated as mechanism(s) of matrine action. Our studies may also be useful to provide more options for neurologists and enable more MS patients to benefit from combination therapy in the future.

## Data Availability Statement

The datasets used in this manuscript can be found in GEO (https://www.ncbi.nlm.nih.gov/), GSE108000.

## Ethics Statement

The animal study was reviewed and approved by the Ethics Committee of Zhengzhou University.

## Author Contributions

LZ designed and supervised the research. MD, XZ, LL and WW conducted in-depth analysis of the selected data set and predicted the possible targets of matrine. MD, XZ, MZ, and MW performed the animal experiments. MD, XZ, LL, and LZ analyzed the data. MZ, YJ, RM and JZ contributed to the reagents, materials, and analysis tools. MD, XZ and LZ wrote the manuscript. All authors contributed to the article and approved the submitted version.

## Funding

This research was supported by the National Natural Science Foundation of China (No.31870334) and the Natural Science Foundation of Henan Province (No.182300410330).

## Conflict of Interest

The authors declare that the research was conducted in the absence of any commercial or financial relationships that could be construed as a potential conflict of interest.
